# Distal shunt placement in pediatric ventriculoperitoneal shunt surgery: an international survey of practice

**DOI:** 10.1007/s00381-023-05855-x

**Published:** 2023-02-13

**Authors:** Linus Ruf, Ladina Greuter, Raphael Guzman, Jehuda Soleman

**Affiliations:** 1 Faculty of Medicine, University of Basel, Basel, Switzerland; 2 Department of Neurosurgery, University Hospital of Basel, Spitalstrasse 21, 4031 Basel, Switzerland; 3 Division of Pediatric Neurosurgery, University Children’s Hospital of Basel (UKBB), Spitalstrasse 21, 4031 Basel, Switzerland

**Keywords:** Ventriculoperitoneal shunt, Ventriculoperitoneal shunt surgery, Pediatric neurosurgery, International survey, Laparoscopy, Mini-laparotomy

## Abstract

**Objective:**

Ventriculoperitoneal shunt (VPS) surgery is a common treatment for hydrocephalus in children and adults, making it one of the most common procedures in neurosurgery. Children being treated with a VPS often require several revisions during their lifetime with a lifetime revision rate of up to 80%. Several different techniques exist for inserting the distal catheter, while mini-laparotomy, trocar, or laparoscopy is traditionally used. As opposed to adults, only few studies exist, comparing the outcome of the different distal catheter placement techniques in children. This international survey aims to investigate the current daily practice concerning distal shunt placement techniques in children.

**Material and methods:**

An online questionnaire investigating the different techniques used to place the distal catheter in pediatric VPS surgery was distributed internationally. All results were analyzed using descriptive and comparative statistics.

**Results:**

A total of 139 responses were obtained. Mini-laparotomy was reported to be the most frequently used technique (*n* = 104, 74.8%) for distal shunt placement in children, while laparoscopic or trocar-assisted placements were only used by 3.6% (*n* = 5) and 21.6% (*n* = 30) of all respondents, respectively. Over half (*n* = 75, 54.0%) of all respondents do not believe that laparoscopic placement improves the outcome.

**Conclusion:**

This international survey shows that mini-laparotomy is the most frequently used technique for distal VPS placement in children all over the world. Further randomized trials are needed to elucidate this matter.

## Introduction

Ventriculoperitoneal shunt (VPS) surgery is a frequent treatment for hydrocephalus in children and adults, making it one of the most common procedures in neurosurgery [1]. Other methods like ventriculoatrial or ventriculopleural shunts are used less frequently due to their higher risk of complications [2, 3]. Many patients, but especially children, with a VPS require revision surgery at some point during their lifetime, which leads to a high socioeconomic burden [4]. Children have been shown to suffer from up to 30–40% [3, 5–7] of shunt failure requiring surgery in the first year, and up to 84.5% requires a revision surgery in a long term [8]. Distal shunt failure rates make up to approximately 30% of all shunt revisions [9, 10]. Traditionally, the distal end of the catheter is placed intraperitoneally via a mini-laparotomy or trocar insertion; however, several studies, most of them conducted in adults, have shown that laparoscopic placement of the distal catheter leads to fewer complications and lower shunt misplacement rates compared to traditional, open placement [11–15]. While there is high-quality evidence for laparoscopic shunt placement in adults, data is scarce in the pediatric population. The aim of this survey was to investigate the current international practice concerning shunt placement techniques in children focused on distal placement techniques.

## Methods

In December 2020, an online survey was distributed through Neurosurgery Research Listserv and the European Association of Neurosurgical Societies (EANS). After 8 weeks, a reminder was sent out again and the survey was closed at the end of April 2021. The questions were prepared and launched via Google Forms (Google LLC, Mountain View, CA, USA), which is a web-based survey platform.

It included a total of 23 questions, of which nine were demographic questions and the rest were focused on surgical techniques (Table 1). The questionnaire was focused on distal shunt placement techniques, but also included questions regarding the proximal catheter placement for comprehensiveness. The survey was considered completed when at least 90% of the questions were answered. Countries were either labeled as high-income, middle-income, or low-income according to the definition by the World Bank [16]. At the end of the survey, all respondents could provide their names if they wanted to be acknowledged for their participation.

**Table 1:** Questions distributed via the online survey

Demographics	
1. What is your gender?	Male/female/prefer not to say
2. What kind of institution do you work in?	University hospital/district hospital/private clinic/other
3. How many years of neurosurgical experience do you have?	1–5/6–10/11–15/16–20/ > 20
4. What function do you have in your department?	Resident/fellow/attending/vice chairman/chairman
5. Are you a fellowship-trained pediatric neurosurgeon?	Yes/no/prefer not to say
6. In which country do you practice neurosurgery?	List of all countries
7. How many ventriculoperitoneal shunt surgeries in children are approximately performed at your institution every year?	< 20/20–40/40–60/60–80/80–100/ > 100
8. How many ventriculoperitoneal shunt surgeries in adults are approximately performed at your institution every year?	< 20/20–40/40–60/60–80/80–100/ > 100
9. Do you operate solely on children?	Yes/no
Shunt placement	
10. What is your most frequently used technique for distal shunt placement?	Open laparotomy/laparoscopy/trocar
11. What is the main reason for your chosen technique?	Evidence in literature/pathology of patient/institute standard/it depends on the available staff/personal opinion/other
12. What is your most frequently used location for proximal shunt placement?	Right frontal/left frontal/right trigonal/left trigonal
13. What is the main reason for your chosen technique?	Evidence in literature/pathology of patient/institute standard/it depends on the available staff/personal opinion/other
14. How do you place your proximal shunt?	Freehand (anatomical landmarks)/ultrasound-guided/navigation-guided
15. Do you use the same techniques also for adults in your hospital?	Yes/no
16. If no, please state what you do differently in adults	Open text
17. If you are performing a laparoscopy for distal shunt placement, who is the laparoscopy done by?	Neurosurgeon/pediatric surgeon
18. Do you believe a better outcome is achieved when the distal shunt is placed via laparoscopy than laparotomy?	Yes/no/maybe
19. Do you believe a better outcome is achieved when the distal shunt is placed via trocar than laparotomy?	Yes/no/maybe
20. What is approximately your shunt revision rate?	< 1%/1–2%/3–5%/6–8%/9–10%/ > 10%
21. In case of revision surgery, do you use the same distal shunt placement technique?	Always/sometimes/never
22. If your answer is “never” or “sometimes,” please explain what you change?	Open text
23. Do you think further studies are necessary investigating shunt placement techniques in children?	Yes/no/maybe

Data was collected automatically through the online platform and exported for analysis. Descriptive statistical analysis was performed using R (R statistical software, Vienna, Austria, version 1.4.1106). Comparable contingency statistics were conducted using Fisher and chi-square tests. For contingency statistics, we dichotomized the answers by the different continents and socioeconomic regions (income group). Furthermore, we added correlations between the proximal and distal placement locations. A *p* value < 0.05 was considered statistically significant.

## Results

### Demographics

A total of 139 neurosurgeons participated in this survey, of which 82.6% (*n* = 114) were male. All participants completed the survey. Nearly half of all participants (*n* = 57, 41.0%) have more than 20 years of experience, and 59.9% (*n* = 82) work as an attending at their institution (Table 2). Over half of the respondents practice neurosurgery in Europe (*n* = 83, 60.6%). A third (*n* = 47, 34.1%) of the participating neurosurgeons operate solely on children. An overview of all demographic data is shown in Table 2.

**Table 2:** Demographic of participating neurosurgeons

	*n* (%)	*N* (total) of replies (139)
**Gender**		
Female	22 (15.9)	138
Male	114 (82.6)	
Prefer not to say	2 (1.4)	
**Institution**		
District hospital	14 (10.1)	139
Private hospital	12 (8.6)	
University hospital	117 (81.3)	
**Years of neurosurgical experience**		
1–5	14 (10.1)	139
6–10	21 (15.1)	
11–15	28 (20.1)	
16–20	19 (13.7)	
> 20	57 (41.0)	
**Fellowship in pediatric neurosurgery**		
Yes	83 (60.1)	138
No	54 (39.1)	
Prefer not to say	1 (0.7)	
**Position**		
Chairman	15 (10.9)	137
Vice chairman	23 (16.7)	
Attending	82 (59.9)	
Fellow	10 (7.3)	
Resident	7 (5.1)	
**Number of VPS in children per year**		
< 20	20 (14.4)	139
20–40	48 (34.5)	
40–60	22 (15.8)	
60–80	18 (12.9)	
80–100	14 (10.1)	
> 100	17 (12.2)	
**Number of VPS in adults per year**		
< 20	57 (41.9)	136
20–40	27 (19.9)	
40–60	21 (15.4)	
60–80	9 (6.6)	
80–100	11 (8.1)	
> 100	11 (8.1)	
**Continent**		
Africa	5 (3.6)	137
America	11 (8.0)	
Asia	24 (17.5)	
Europe	83 (60.6)	
South America	14 (10.2)	
**Gross national income**		
High-income country	96 (70.1)	137
Middle-income country	38 (27.7)	
Low-income country	3 (2.2)	

### Surgical technique

#### Distal placement

A majority of the participating neurosurgeons use a mini-laparotomy as their preferred technique to place the distal shunt catheter (*n* = 104, 74.8%), followed by placement via a trocar (*n* = 30, 21.6%), while only a minority (*n* = 5, 3.6%) of respondents stated to use laparoscopy as their standard technique (Table 3). Nearly all respondents (*n* = 107, 79.3%) use the same technique in children and adults. Nearly half (*n* = 74, 48.0%, multiple answers possible) stated that the main reason for them to place the distal catheter via a mini-laparotomy is due to an institutional standard. Most of the respondents do not believe that the different distal insertion techniques would change the outcome (no difference between laparoscopy [*n* = 75, 54.0%] and trocar [*n* = 48, 65.8%] compared to mini-laparotomy, Table 3).

**Table 3:** Surgical techniques for primary insertion and revision surgery

	*n* (%)	*N* (total) replies
**Technique for distal catheter**		
Laparoscopy	5 (3.6)	139
Mini-laparotomy	104 (74.8)	
Trocar	30 (21.6)	
**Standard location for proximal shunt**		
Right trigonal	76 (54.7)	139
Right frontal	63 (45.3)	
**Placement technique for proximal shunt (multiple answers possible)**		
Freehand (anatomical landmarks)	104 (65.0)	160
Navigation-guided	35 (21.9)	
Ultrasound-guided	17 (10.6)	
Other	4 (2.5)	
**In case of laparoscopy, surgeon performing it (main technique or revision surgery)**^**a**^		
Neurosurgeon	26 (25.2)	103
Pediatric surgeon	68 (66.0)	
Not sure/not performed at their institution	9 (8.7)	
**Same technique adults and children**		
Yes	107 (79.3)	135
No	28 (20.7)	
**Same technique in case of revision**		
Always	83 (59.7)	139
Sometimes	56 (40.3)	
Never	0 (0)	
**Assumed better outcome with laparoscopy**		
Yes	14 (10.1)	139
Maybe	50 (36.0)	
No	75 (54.0)	
**Assumed better outcome with trocar**		
Yes	8 (11.0)	73
Maybe	17 (23.3)	
No	48 (65.8)	
**Necessity of further studies**		
Yes	80 (57.6)	139
No	22 (15.8)	
Maybe	37 (26.6)	

^a^The answers might refer to revision surgery, in which the neurosurgeon changed to a laparoscopic approach, or they might have understood it as a hypothetical question

In case of a laparoscopic insertion, two-thirds of the respondents (*n* = 68, 66.0%) would perform it together with a pediatric surgeon. Most participants believe that more research on this topic is (*n* = 80, 57.6%) necessary.

#### Proximal placement

Right trigonal shunt placement was the preferred location for proximal shunt catheter placement (*n* = 76, 54.7%) followed by right frontal placement (*n* = 63, 45.3%), while none of the respondents uses the left side as the standard approach (Table 3). This approach was chosen due to an institute’s standard in 37.1% (*n* = 65), followed by the respondents’ personal preference in 29.1% (*n* = 51, multiple answers possible). The majority (*n* = 104, 65.0%) of the participating neurosurgeons place the catheter freehand with anatomical landmarks, followed by navigation (*n* = 35, 21.9%) and ultrasound guidance (*n* = 17, 10.6%, Table 3).

#### Revision surgery

**Fig. 1:**
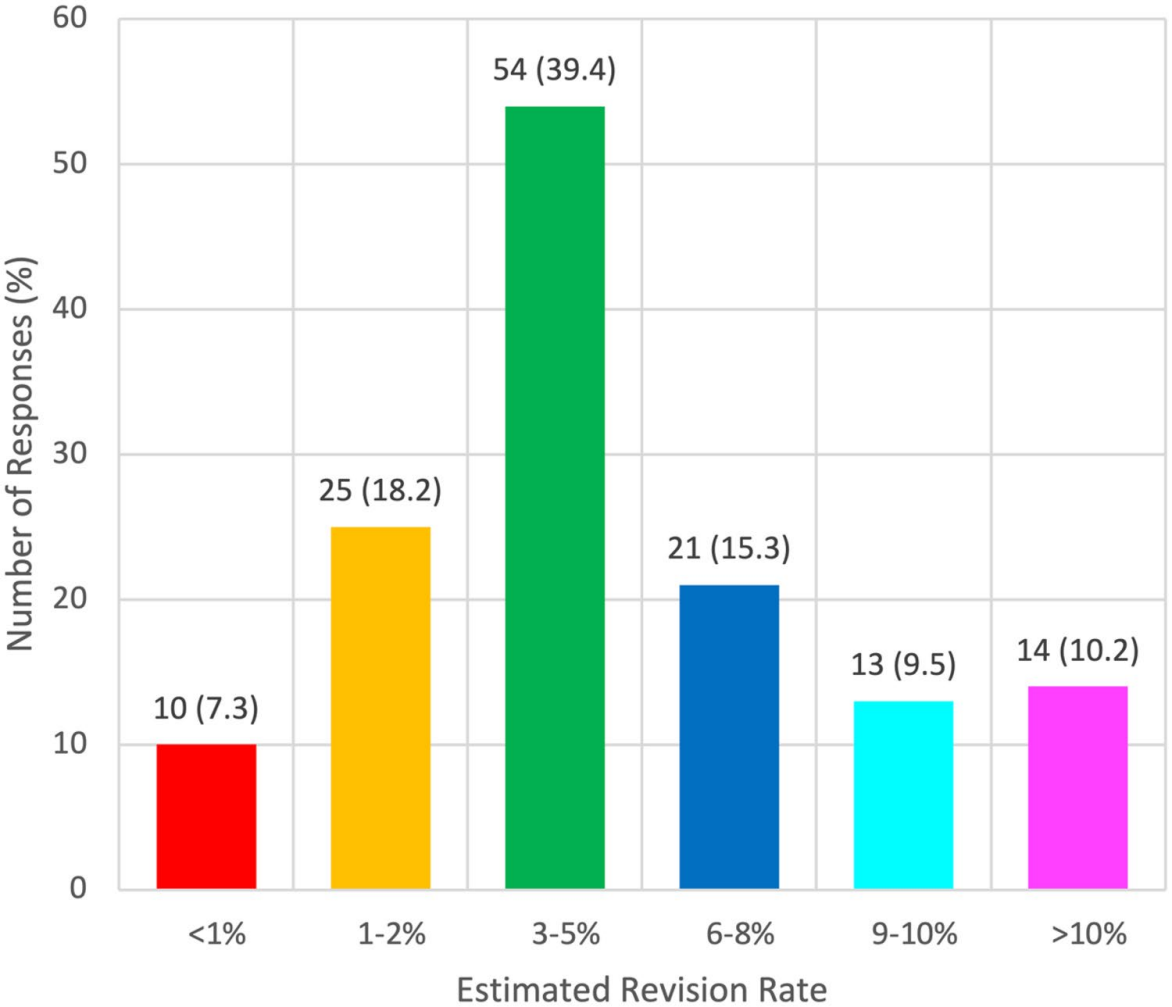
Bar chart showing the estimated shunt revision rates among respondents

An estimated shunt revision rate of 3–5% was reported by 39.4% (*n* = 54) of all respondents (Fig. 1).

In case of a VPS revision, 59.7% (*n* = 83) of all respondents use the same technique as they used for the initial distal placement, while the remaining (*n* = 56, 40.3%) stated to adapt their technique depending on the type of shunt failure and specific patient characteristics (Table 3).

### Analysis of influencing parameters

 Comparing the answers according to the different countries’ socioeconomic groups, no difference could be detected for distal shunt placement techniques (*p* = 0.207, Table 4). However, high-income countries place the proximal catheter significantly less often right trigonal compared to middle- or low-income countries (44.8% vs. 76.3% vs. 66.7%, *p* = 0.004, Table 4). They also use significantly more often navigation (28.7% vs. 5.0% vs. 0.0%, *p* = 0.006, Table 4) or ultrasound guidance (13.0% vs. 5.0% vs. 0.0%, *p* = 0.006, Table 4) compared to middle- or low-income countries. The same difference can be detected between the continents. The respondents in North America and Europe use significantly more often navigation (27.6% and 31.2% vs. 0.0% vs. 0.0% vs. 11.5%, *p* = 0.043, Table 5) or ultrasound guidance (15.3% and 6.2% vs. 0.0% vs. 0.0% vs. 3.8%, *p* = 0.043, Table 5) compared to Africa, South America, and Asia.

**Table 4:** Shunt placement techniques according to the different income classifications of countries

	High-income	Middle-income	Low-income	*p* value
	*n* = 96	*n* = 38	*n* = 3	
**Technique for distal catheter (%)**				
Laparoscopy	5 (5.2)	0 (0.0)	0 (0.0)	0.207
Mini-laparotomy	67 (69.8)	33 (86.8)	3 (100.0)	
Trocar	24 (25.0)	5 (13.2)	0 (0.0)	
**Standard location for proximal shunt (%)**				
Right frontal	53 (55.2)	9 (23.7)	1 (33.3)	0.004
Right trigonal	43 (44.8)	29 (76.3)	2 (66.7)	
	*n* = 115	*n* = 40	*n* = 3	
**Placement technique for proximal shunt (%)**				
Freehand (anatomical landmarks)	63 (54.8)	36 (90.0)	3 (100.0)	0.006
Navigation-guided	33 (28.7)	2 (5.0)	0 (0.0)	
Ultrasound-guided	15 (13.0)	2 (5.0)	0 (0.0)	
Other	4 (3.5)	0 (0.0)	0 (0.0)

**Table 5:** Shunt placement techniques according to the different continents

	Africa	Europe	North America	South America	Asia	*p* value
	*n* = 5	*n* = 83	*n* = 11	*n* = 14	*n* = 24	
**Technique for distal catheter (%)**						
Laparoscopy	0 (0.0)	4 (4.8)	1 (9.1)	0 (0.0)	0 (0.0)	0.009
Mini-laparotomy	5 (100.0)	61 (73.5)	3 (27.3)	12 (85.7)	22 (91.7)	
Trocar	0 (0.0)	18 (21.7)	7 (63.6)	2 (14.3)	2 (8.3)	
**Standard location for proximal shunt (%)**						
Right frontal	2 (40.0)	45 (54.2)	8 (72.7)	3 (21.4)	5 (20.8)	0.005
Right trigonal	3 (60.0)	38 (45.8)	3 (27.3)	11 (78.6)	19 (79.2)	
	*n* = 5	*n* = 98	*n* = 16	*n* = 13	*n* = 26	
**Placement technique for proximal shunt**						
Freehand (anatomical landmarks)	5 (100.0)	53 (54.1)	9 (56.2)	13 (100.0)	22 (84.6)	0.043
Navigation-guided	0 (0.0)	27 (27.6)	5 (31.2)	0 (0.0)	3 (11.5)	
Ultrasound-guided	0 (0.0)	15 (15.3)	1 (6.2)	0 (0.0)	1 (3.8)	
Other	0 (0.0)	3 (3.1)	1 (6.2)	0 (0.0)	0 (0.0)	

Significantly more responders from North America and Europe place the distal shunt via laparoscopy compared to Africa, Asia, and South America (9.1% and 4.8% vs. 0% for the rest, *p* = 0.009, Table 5).

## Discussion

Based on this international survey with 139 participants, most of the respondents (*n* = 104, 74.8%) use a mini-laparotomy for distal shunt insertion in children. No difference for distal VPS placement techniques between the different socioeconomic regions could be detected.

Although over half of the participants (*n* = 75, 54.0%) do not believe in a better outcome using laparoscopy over mini-laparotomy, most (*n* = 80, 57.6%) think that further studies on this topic are necessary.

### Distal placement

Traditionally, distal VPS insertion is performed by an open mini-laparotomy. However, several studies in adults have recently proven that laparoscopic VPS insertion can significantly reduce the risk of distal shunt failures due to abdominal malpositioning, and shorter operating time [12, 17, 18]. Phan et al. [15] showed in their systematic review and meta-analysis that laparoscopic VPS placement has a significantly lower rate of distal obstruction and malfunction; however, this data is only limited to adults. In the pediatric population, the data is scarce, consisting mainly of retrospective cohort analysis. These studies showed that laparoscopic VPS placement is safe in children and a possible alternative to the traditional mini-laparotomy and suggest an improvement in outcome [1, 9, 10, 19–23]. Laparoscopy has been shown to have very low failure rates, with some studies reporting no distal failure [21, 24]. However, some studies such as the study by Yu et al. [9] also included a pre-selected subset of pediatric patients with previous shunt malfunctions undergoing revision surgery. In their study, 45% of patients were not safely amenable to laparoscopic insertion due to extensive adhesions and had to be converted to an open laparotomy or even to a ventriculopleural shunt. In such a subgroup of pediatric patients, the risks and complications are increased compared to regular shunt insertions and could skew the risk perception of laparoscopic shunt placement in children [9]. Moreover, historically, laparoscopy in children < 1 year used to be considered too dangerous due to their thin abdominal wall, thin tissue, and low weight (< 5 kg) [1, 10]. However, two studies could show that laparoscopic VPS insertion is safe in children < 1 year and below the weight of 5 kg [22, 23]. The main goal of laparoscopic VPS placement is to reduce complications, especially distal catheter misplacement, and failure rates and improve surgical safety. The main advantage of laparoscopy compared to mini-laparotomy is that the shunt tip can be placed under vision into the peritoneum and that it leads to a smaller incision and smaller opening of the peritoneum. Furthermore, possible adhesions, leading to shunt dysfunction, are more readily detected and the shunt can be optimally placed. Several studies describe the successful use of laparoscopic shunt insertion for complex and complicative cases after several revisions or abdominal surgery [10, 20, 25–29]. In our survey, 59.7% (*n* = 83) of all participating neurosurgeons would use the same method again for revision surgery; however, most of them also mentioned they would consider changing their technique depending on the type of shunt failure and individual patient history. Moreover, we think the team should perform surgery with the technique they are most familiar with and, in case of laparoscopic surgery in children, especially neonates or high-risk patients, laparoscopy should be performed by an experienced pediatric surgeon [19]. Additionally, laparoscopic VPS insertion can be of advantage in obese patients, as a mini-laparotomy in these patients is challenging, often resulting in a larger incision and difficulties identifying the different anatomical layers [18]. This is important as the prevalence of obese children and young adults is rising, resulting in pediatric neurosurgeons being more often confronted with these patients [30].

Another commonly used VPS insertion technique is by using a trocar. In our survey, 21.6% (*n* = 30) of respondents reported using this as their standard technique. This technique uses a single trocar, which is blindly introduced into the peritoneal cavity. There is only one comparative study, published by our group, comparing trocar-assisted to laparoscopic distal VPS placement. Trocar-assisted placement had a non-statistically significantly higher rate of distal malfunction and distal complications compared with laparoscopic-guided shunt placement [14].

In the present survey, a significant difference between the continents for the placement technique of the distal catheter (*p* = 0.009) could be detected; however, due to the low number (*n* = 5) of neurosurgeons who primarily insert VPS laparoscopically, a bias cannot be avoided. Furthermore, most of the participants practice neurosurgery in Europe, which could skew the results and might only reflect the current practice in this region. In general, laparoscopic surgery is less often used in low- and middle-income countries. Studies have identified several causes for this, such as lack of equipment and personnel, as well as sociocultural barriers, which might explain the differences we observed between the different socioeconomic regions in our survey [31, 32].

### Proximal placement

According to the results of this survey, the difference between proximal catheter placement in the right trigonum or the frontal horn is marginal (*n* = 76, 54.7% vs. *n* = 63, 45.3%). It has been widely debated whether trigonal shunt placement has a higher risk of failure compared to frontal placement, mainly due to the proximity of the choroid plexus, which could cause obstruction of the proximal catheter. However, no clear consensus exists, which is reflected in the responses given in this survey [8, 33–37].

Most of the respondents (*n* = 104, 65.0%) preferred freehand shunt placement, followed by navigation (*n* = 35, 21.9%) and ultrasound (*n* = 17, 10.6%) guidance. Recently, several studies have shown that freehand shunt placement is significantly associated with a higher shunt misplacement rate and higher early revision rate compared to either navigation or ultrasound-guided placement [38–42]. These answers in this survey do not reflect the findings in the current literature; however, this could be due to the recent nature and the quality of these studies.

## Limitations

This study is a survey and is inherent to all limitations of such a study. Self-reported data represents an unavoidable limitation. Moreover, the response rate might be low with a total of 139, which could introduce a certain bias and might not be representative of the whole neurosurgical community. Most of the participating neurosurgeons practice in Europe, limiting the results to this socioeconomic region and might not be reflecting the practice in other continents especially in low-income countries.

## Conclusion

To our knowledge, this is the first international survey investigating the practice of distal shunt placement in children. According to the literature and our survey, mini-laparotomy is overall still the standard technique for distal shunt placement in children; however, laparoscopy seems to have advantages especially in patients with previous abdominal surgery. Most of the respondents do not believe that the distal VPS placement technique influences the outcome; however, they agree that more research investigating this is necessary.

## Data Availability

The data that support the findings of this study are available from the corresponding author, upon reasonable request.
